# Targeting Tumor-Associated Macrophages in Cancer Immunotherapy

**DOI:** 10.3390/cancers13215318

**Published:** 2021-10-22

**Authors:** Amy J. Petty, Dwight H. Owen, Yiping Yang, Xiaopei Huang

**Affiliations:** 1 Department of Medicine, Duke University Medical Center, Durham, NC 27710, USA; amy.petty@duke.edu; 2 Division of Medical Oncology, Department of Internal Medicine, College of Medicine and OSU Comprehensive Cancer Center, The Ohio State University, Columbus, OH 43210, USA; Dwight.owen@osumc.edu; 3 Division of Hematology, Department of Internal Medicine, College of Medicine and OSU Comprehensive Cancer Center, The Ohio State University, Columbus, OH 43210, USA; Yiping.yang2@osumc.edu

**Keywords:** tumor-associated macrophages (TAM), tumor microenvironment, immunotherapy, checkpoint inhibitor, cancer

## Abstract

**Simple Summary:**

Tumor-associated macrophages (TAMs) coinhabit the tumor microenvironment with cancer, immune, and stromal cells. They undermine the immune system and facilitate tumor growth and metastasis. In this review, we discussed current understanding of TAMs functions, and strategies harnessing the knowledge gained from recent research to develop innovative cancer treatments. We summarized pre-clinical/clinical studies targeting TAMs with small molecule inhibitors or antibodies alone or combined with chemotherapy/immunotherapy, evaluated the efficacy of these therapies, and discussed mechanisms of actions.

**Abstract:**

Tumor-associated macrophages (TAMs) represent the most abundant leukocyte population in most solid tumors and are greatly influenced by the tumor microenvironment. More importantly, these macrophages can promote tumor growth and metastasis through interactions with other cell populations within the tumor milieu and have been associated with poor outcomes in multiple tumors. In this review, we examine how the tumor microenvironment facilitates the polarization of TAMs. Additionally, we evaluate the mechanisms by which TAMs promote tumor angiogenesis, induce tumor invasion and metastasis, enhance chemotherapeutic resistance, and foster immune evasion. Lastly, we focus on therapeutic strategies that target TAMs in the treatments of cancer, including reducing monocyte recruitment, depleting or reprogramming TAMs, and targeting inhibitory molecules to increase TAM-mediated phagocytosis.

## 1. Introduction

Macrophages represent one of the largest leukocyte populations within the tumor stroma and play an important role in neoplastic progression. These macrophages, also known as tumor-associated macrophages (TAMs), can promote tumor cell growth through a variety of mechanisms including enhanced angiogenesis and chemotherapy resistance as well as by suppressing anti-tumor immunity [1,2]. Increased infiltration of macrophages is correlated with poor clinical prognosis in many cancers [2]. This review focuses on the role of TAMs in tumor progression and immunotherapeutic options to target this population. 

## 2. Macrophage and TAMs

Macrophages play important roles in the human body, ranging from tissue homeostasis to protection against pathogenic infections. Various environmental signals can activate a broad array of intracellular transcriptional networks in macrophages resulting in the polarization of functions. The M1/M2 polarization states of macrophages include the classically activated M1 macrophages and the alternatively activated M2 macrophages [3]. Though these states mirror the T helper 1 (Th1)-Th2 states of T cells, the M1/M2 phenotypes are not stably differentiated in the same way as Th1 and Th2 cells. Rather, macrophages in human patients as well as mouse models can exhibit phenotypes in a spectrum with two extremes defined by M1 and M2 [4]. Interferon-γ (IFN-γ), microbial products (e.g., lipopolysaccharides/LPS), granulocyte-macrophage colony stimulating factor (GM-CSF), and Th1 cytokines such as interleukin-12 (IL-12) activate M1-polarization of macrophages, which are involved in innate host defense and pro-inflammatory functions by expressing high levels of cytokines such as IL-1, IL-6, and IL-12; tumor necrosis factor (TNF); surface receptors such as Fcy-RI, II, and IIIA; and reactive oxygen/nitrogen species (ROS/RNS) [3]. In contrast, M2 macrophages are activated by IL-4, IL-13, IL-10, and macrophage colony-stimulating factor (M-CSF/CSF-1) and in turn produce large amounts of anti-inflammatory, pro-fibrotic, and pro-angiogenic cytokines such as IL-10, transforming growth factor-beta (TGF-β), vascular endothelial growth factor (VEGF), and matrix metalloproteins (MMPs) [3,5]. M1 and M2 polarized macrophages both play an important role in tissue homeostasis and tissue recovery with M1 macrophages infiltrating after injury to clean the wound of bacteria, debris, and dead cells, and M2 follows to promote anti-inflammatory effects and restoration of local structure and vasculature through the actions of associated cytokines. In chronic wounds, pro-inflammatory M1 macrophages persist without transitioning to M2 macrophages, which is a necessary step in tissue repair, ultimately resulting in impairment in wound healing [6]. Although these cytokines and molecules can be beneficial in normal tissue healing and remodeling, they may enhance tumor progression within the tumor microenvironment. Furthermore, M1 and M2 macrophages are associated with different chemokine profiles. M1 macrophages produce high levels of CXCL9, CXCL10, and CCL5 that can promote cytotoxic T cell functions with host defense and in tumor. On the other hand, M2-associated chemokines including CCL17 and CCL22 help recruit regulatory T cells (T_reg_) that can further dampen host immune responses at the tumor site [5]. Interestingly, CCL2 has been implicated in both M1 and M2 polarization. There are observations that direct stimulation of cells with CCL2 favored M1 macrophage polarization, and that CCL2 or CCR2 knockout mice showed decreased M1 markers including iNOS, IL-12 and TNF-α compared to wildtype unstimulated bone marrow derived macrophages [7,8,9]. However, in cancer, strong evidence indicates that CCL2-CCR2 blockade suppresses M2 polarization and enhances T cell cytotoxicity [10]. There are ongoing debates about the dichotomous and highly context specific roles of CCL2 in benign vs. malignant inflammatory processes. Further investigation in this area is needed to guide the development of therapies targeting CCL2-CCR2 in cancer. 

Though often considered interchangeable with M2-macrophages, TAMs should be considered a separate group of pro-tumoral macrophages due to their distinct transcriptional and phenotypic characteristics [11]. The simplified M1-M2 dichotomy does not consider a variety of factors that can potentially influence TAM functions, including location, type, and stage of the tumor. It has been a challenge to identify specific subgroups of TAMs with overlapping features based on one set of specific markers due to intrinsic heterogeneity of the macrophage population. Additionally, it remains unanswered whether the heterogeneity observed in TAMs stem from distinct precursors or variance in microenvironmental stimuli during tumor development. 

In addition to difficulties encountered in classifying TAMs in vivo, the origin of TAMs also remains controversial. It has been hypothesized that TAMs are infiltrating monocytes recruited from circulation via chemotaxis. Studies have shown that TAMs are replaced by circulating Ly6C^hi^CCR2^+^ monocytes in various tumor models [12,13,14,15,16]. Circulating inflammatory Ly6C^hi^CD11c^−^MHCII^−^CD11b^hi^CCR2^+^ monocytes, derived from BM hematopoietic stem cells are recruited to tumor sites and later differentiated into TAMs. A study by Franklin RA et al., using a PyMT mammary tumor model suggested a key role of Notch signaling pathway in TAM differentiation [13]. In addition, using mouse models that enable BM-derived monocytes tracking, Shand et al. showed that tumor-associated macrophages were derived and rapidly replenished from the BM in tumor-bearing mice [17]. Others have shown that tissue-resident macrophages may play an equally important role in contributing to the TAM population. In non-small cell lung cancer (NSCLC), tissue resident macrophages provide a pro-tumorigenic niche to early NSCLC cells [18]. Zhu et al. identified both inflammatory monocytes and tissue-resident macrophages as sources of TAMs with monocyte-derived TAMs playing more important roles in antigen presentation and embryonic-derived TAMs exhibiting a more pro-fibrotic profile [19]. Further studies are needed to examine the potentially different roles of monocytic vs. embryonic-derived TAMs in tumor development and progression. Nonetheless, TAM expansion and polarization within the tumor microenvironment requires myeloid cell recruitment and factors that drive the immunosuppressive polarization of TAM. Increased levels of CCL2, CSF1, and VEGF-A are correlated with macrophage accumulation at the tumor site in a wide spectrum of human cancers, including those of breast, prostate, ovarian and lung [20,21,22]. Once recruited to the primary tumor site, various signaling pathways, including JAK/Stat, PI3K/Akt, and hedgehog orchestrate the polarization under the influence of various tumor-derived or stroma-derived factors [23,24]. IL-4 and IL-13, synthesized by CD4^+^ T cells and/or tumor cells act on infiltrating macrophages through intracellular Stat6 and PI3K signaling to promote an immunosuppressive TAM phenotype while IL-10 produced by regulatory T cells (T_reg_) also participates in the activation of the TAM phenotype via the actions of Stat3. Other cytokines secreted by the tumor cells, including CSF1 and TGF-β also strongly promote M2-polarization of TAMs [23,25]. Lactic acid, a tumor cell-derived aerobic glycolysis byproduct induces TAM functional polarization through the action of hypoxia inducible factor 1α (HIF-1α) resulting in subsequent promotion of tumorigenesis [26]. In our laboratory, we demonstrated that tumor-derived sonic hedgehog (Shh) ligand promotes M2-polarization of TAMs to suppress CD8^+^ T cell infiltration through downregulation of CXCL9 and CXCL10 and limit effector T cell functions by upregulating programmed death ligand-1 (PD-L1) expression on TAMs [24,27]. However, TAM polarization is a dynamic process and demonstrates great functional and phenotypic diversity depending on the cancer type and tumor stage [2]. Understanding the origin of macrophages within the tumor stroma in different types and stages of cancer, and the mechanisms of recruitment, retention, and differentiation is crucial for the design of rational strategies to effectively targeting this population of cells in cancer immunotherapy. 

## 3. The Functions of Tumor-Associated Macrophages

**Figure 1:**
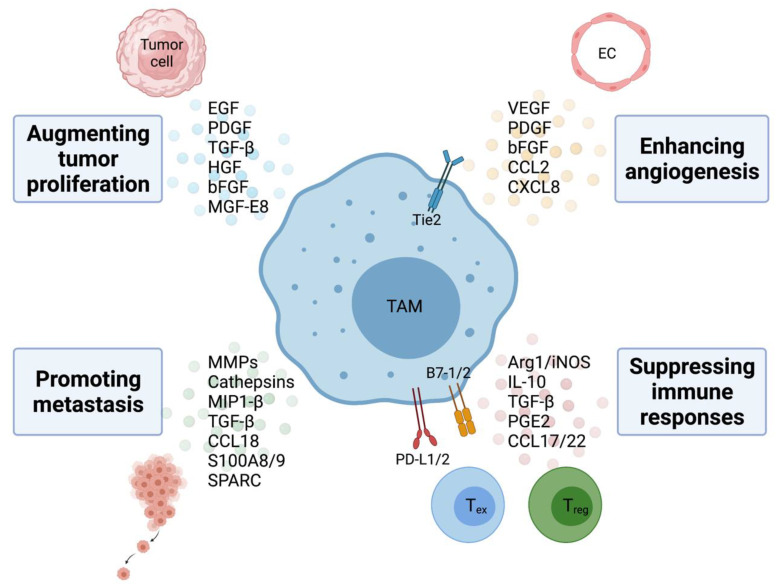
Functional roles of TAMs. TAMs can promote tumor progression through four main mechanisms: (1) augmenting tumor proliferation; (2) enhancing angiogenesis; (3) promoting metastasis; and (4) suppressing adaptive immune responses. Abbreviations: EGF—epithelial growth factor; PDGF—platelet-derived growth factor; TGF-β—transforming growth factor beta; HGF—hepatocyte growth factor; bFGF—basic fibroblast growth factor, MGF-E8—milk fat globule-EGF factor 8; EC—endothelial cell; VEGF—vascular endothelial growth factor; CCL2—C motif chemokine ligand 2; CXCL8—C-X-C motif chemokine ligand 8; MMPs—matrix metalloproteases; MIP1-β—macrophage inflammatory protein 1 beta; CCL18—C motif chemokine ligand 18; S100A8/9—S100 calcium binding protein A8/9; SPARC—secreted protein acidic and cysteine rich; Arg1—arginase 1; iNOS—inducible nitric oxide synthase; IL-10—interleukin 10; PGE2—prostaglandin E2; CCL17/22—C motif chemokine ligand 17/22; PD-L1/2—programmed death ligand ½; T_ex_—exhausted T cell; T_reg_—regulatory T cell. Created with BioRender.com.

TAMs within the tumor microenvironment play a pivotal role in promoting tumorigenesis and metastasis via both non-immune and immune mechanisms, leading to poor clinical outcome in patients with various types of solid tumors and hematologic malignancies [28,29]. The critical role of TAM in promoting tumor cell proliferation, angiogenesis, metastasis, and immunosuppression within the TME are discussed below and illustrated in Figure 1.

### 3.1. Augmenting Tumor Proliferation

TAMs not only provide structural support within the tumor stroma but can also facilitate cancer initiation by secreting signal molecules, including growth factors, such as epithelial growth factor (EGF), platelet-derived growth factor (PDGF), TGF-β, hepatocyte growth factor (HGF), and basic fibroblast growth factor (bFGF) among other cytokines and chemokines [30]. Additionally, Tong et al. reported that TAM-derived CXCL8 decreases ERα expression, resulting in endometrial cancer progression [31]. In pancreatic ductal adenocarcinoma, TAM-derived IL-1β prevents expression of 15-hydroxyprostaglandin dehydrogenase (15-PGDH) and low 15-PGDH is correlated with more advanced tumor stage and lymph node metastasis [32]. 

In addition to supporting tumor cell growth, TAMs have also been found to support cancer stem cells (CSCs), a group of cells within the tumor stroma that can proliferate indefinitely, resulting in tumor progression and increased resistance to cytotoxic therapy [33,34]. TNF-α released by TAMs triggers the expansion of a group of stem-like cancer cells via NF-*κ*B signaling in a colon cancer while TAM-derived IL-6 promotes CSCs growth via Stat3 in human hepatocellular carcinoma [35,36]. Akt/mTOR signaling in renal cell carcinoma was also found to increase stem cell-like populations in the presence of TAMs [37]. Additionally, juxtacrine communications between CSCs and TAMs via the CD90 and Eph4A receptors sustain the CSC niche in breast cancer by activating Src and NF-*κ*B [38]. Recent studies also demonstrated that TAM-derived milk fat globule–epidermal growth factor 8 (MFG-E8) favors CSC reservoir survival during chemotherapeutic treatments via activation of Sonic hedgehog and Stat3 pathways [39]. Yang et al. reported that gene signatures representing CSC properties (Sox-2, Oct-4, Nanog, AbcG2 and Sca-1) were found to be upregulated by TAMs via a paracrine EGFR/Stat3/Sox2 signaling pathway, resulting in increased drug efflux capacity and resistance to chemotherapy in murine breast cancer cells [40]. Not surprisingly, inhibiting CSF1R or CCR2 on TAMs arrests the proliferation of CSCs, resulting in improved chemotherapeutic responses in pancreatic carcinoma [41]. 

### 3.2. Enhancing Angiogenesis

Previous evidence has shown that TAMs are extensively involved in each step of the angiogenic process from degradation of the basement membrane through the production of matrix metalloproteinases (MMPs) and cathepsins to the secretion of proangiogenic growth factors such as VEGF, PDGF, bFGF, and chemokines CCL2 and CXCL8 that provide the vascular network critical for cancer cell growth and dissemination [42,43]. Additionally, VEGF and CCL2 also act as strong chemotactic factors for the infiltration and polarization of TAMs and increased expression of both is positively correlated with a high degree of tumor neovascularization in human invasive ductal breast carcinoma [44]. Therefore, TAMs play a critical role in tumor angiogenesis by working in concert with tumor-derived angiogenic factors. 

Interestingly, a unique subset of Tie2^+^ macrophages have been shown to regulate angiogenic switch in the PyMT breast cancer model [45]. Hypoxia and CSF-1 upregulate Tie2 expression on TAMs and then the expression of angiopoietin 1 and 2, ligands for Tie2 on endothelial cells allows for the alignment of Tie2^+^ macrophage along the abluminal surface of blood vessels, resulting in macrophage-synthesized WNT7b targeting of vascular endothelial cells, their production of VEGF and ultimately activation of the angiogenic switch [46,47,48]. Hypoxia itself is also a potent regulator of the pro-angiogenic functions of TAMs via the transcription factor hypoxia-inducible factor 1-α (HIF-1α), which upregulates VEGF expression [49]. Lastly, Weichand et al. also revealed that TAMs signal through the S1PR1/NLRP3/IL-1β cascade to infiltrate into tumors to promote pulmonary metastasis and lymphatic angiogenesis in mouse breast cancer model. Increased NLRP3 expression in TAMs is correlated with lymph node invasion and metastasis in breast cancer patients [50]. 

### 3.3. Promoting Metastasis

Extensive evidence has shown a correlation between TAM recruitment and increased tumor cell invasiveness [51]. TAMs not only contribute to early epithelial–mesenchymal transition (EMT) of tumor cells but also help prepare a distant site primed to support metastatic growth. One of the primary mechanisms TAMs facilitate tumor cell invasion and migration is by secreting MMPs, serine proteases and cathepsins which disrupt cell junctions and basal membrane [52]. Vasiljeva et al. reported that TAM-derived cathepsin B promotes invasion and lung metastasis in PyMT breast cancer model [53]. Additionally, other recent reports also found that TAM-derived macrophage inflammatory protein 1-β and TGF-β can both lead to cancer cell matrix protrusion and invasion in breast cancer and non-small cell lung cancer, respectively [54,55]. TAMs also abundantly produce CCL18 which triggers integrin clustering around human breast cancer cells and allows for their adherence to ECM through interactions with the phosphatidylinositol transfer protein, membrane-associated 3 receptor (PITPNM3), resulting in enhanced intravasation, metastasis and reduced patient survival [56]. TAMs also produce several other molecules that augment cell invasion and metastasis. Lim et al. reported that increased S100A8 and S100A9 secretion from TAMs result in tumor invasion and migration in colon and Lewis lung carcinoma cells [57]. TAM-derived Secreted Protein Acidic and Rich in Cysteine (SPARC) enhances tumor extracellular matrix deposition and interaction [58].

In terms of enhancing EMT, using two solid tumor models, researchers showed that macrophage-derived TGF-β induced EMT-mediating pathway in tumor cells, resulting in increased expression of mesenchymal markers and an invasive phenotype [59,60,61]. Lastly, the identification of a distinct population of VEGFR1^high^CX3CR1^high^CCR2^high^ but Tie2^−^ macrophage at the lung metastatic site in a spontaneous breast cancer model demonstrated that macrophages not only serve to prepare the pre-metastatic niche to increase metastatic efficiency but also foster cancer cell survival and growth at the distant site [62].

### 3.4. Suppressing Adaptive Immune Responses

Substantial evidence has supported that pro-tumorigenic TAMs can subvert tumor-infiltrating T lymphocytes functions directly and further modify the immune cell composition within the TME to decrease antitumoral immune cells while simultaneously increase the presence of immunosuppressive cell types to accelerate tumorigenesis. TAMs contribute greatly to tumor immune evasion by exerting immunosuppression on adaptive immune responses through the secretion of cytokines, chemokines, and enzymes directly or indirectly [63,64]. They serve as crucial drivers of chronic tumor-associated inflammation, and work in concert with tumor cells and other stromal cells in the tumor microenvironment to promote tumor progression.

TAMs suppress CD8^+^ T cell activation via several major mechanisms: (1) interference of CD8 T cell trafficking to the tumor site; (2) depletion of metabolites essential for T cell proliferation; (3) secretion of anti-inflammatory cytokines; and (4) activation of T cell checkpoint blockade. Peranzoni et al. showed that depletion of TAMs using a CSF-1 receptor inhibitor enhances CD8 T cell migration and infiltration into tumor islets [65]. Our laboratory further demonstrated that hedgehog signaling in hepatocellular carcinoma promotes the immunosuppressive M2 phenotype of TAMs, which further impedes CD8^+^ cytotoxic T lymphocyte trafficking into the tumor stroma via suppression of CXCL9 and CXCL10 [24]. Additionally, metabolism of L-arginine and L-tryptophan, catalyzed by TAM-derived arginase-1 and indoleamine dioxygenase 1/2 (IDO 1/2), respectively, results in the failure to re-express CD3 ζ-chain in the T cell receptor (TCR) complex and inability to respond to tumor antigen [66,67,68]. CD8^+^ T cells are also highly sensitive to the L-arginine level as it is required for the development of memory T cells which confers longer lasting cancer immunity [69]. Another power regulator of T cell suppression is tissue hypoxia, which augments the levels of HIF-1α and HIF-2α in TAMs. Higher levels of HIF-1α and HIF-2α can subsequently upregulate Arg1 and iNOS levels to exhaust L-arginine and increase NO in the TME [70]. Furthermore, anti-inflammatory cytokines, including IL-10, TGF-β, prostaglandin-E2 (PGE_2_) produced by TAMs can inhibit cytotoxic CD8^+^ and helper CD4^+^ cells to establish an overwhelmingly immunosuppressive TME [63,71,72]. TAMs are the primary source of cytokine IL-10 within the tumor microenvironment such as in mammary carcinomas, and can indirectly blunt CD8^+^ T cell responses by inhibiting DC production of IL-12 or directly on CD4^+^ T cells, inhibiting proliferation and production of IL-2, IFN-γ, and TNF-α, resulting in suppressing proinflammatory responses within the tumor microenvironment [63]. TAMs also express TGF-β, a cytokine with multiple immunosuppressive properties in various tumor models. These properties include inhibition of T-cell proliferation, inhibition of T-cell differentiation into cytotoxic T lymphocytes (CTLs) and helper T cells, and inhibition of the T-cell stimulatory functions of APCs [73]. IL-10 and TGF-β have been implicated in promoting T cell exhaustion, characterized by the loss of effector functions and the inability to transition into memory T cells, both in models of chronic viral infections and cancer [74]. Lastly, TAMs can suppress the function of tumor-infiltrating lymphocytes (TILs) by expressing the ligands of the inhibitory receptors PD-1 and CTLA-4 [75,76]. When activated by ligands, PD-L1, PD-L2 and B7-1 (CD80), B7-2 (CD86), PD-1, and CTLA-4, respectively, inhibit downstream TCR and BCR signaling, T cell activation, proliferation, and cytotoxic functions. Our laboratory found that tumor-derived Shh ligand promotes PD-L1 expression on TAMs in a mouse hepatocellular carcinoma model and higher expression of PD-L1 results in suppressed production of IFN-γ and granzyme-B in CD8+ TILs [27]. Another challenge previously encountered was to decipher the relative contribution of TAM-expressed PD-L1 on T cell suppression in the TME as tumor cells and various other cell types also express PD-L1 [77]. Our recent study provided definitive evidence that TAM-expressed PD-L1 plays a major role in suppressing TILs using a CD274/PD-L1 conditional knockout mouse model [27]. 

In addition to suppressing anti-tumorigenic effector cells, TAMs also help attract or induce immunosuppressive cells at the tumor site. T_reg_ cells, a subset of CD4^+^ T cells characterized by its ability to dampen immune responses, are attracted to the tumor stroma via chemokine receptors CCR4, CCR5, CCR6, and CCR10 [78]. TAMs in ovarian and colorectal cancers produce CCL17/CCL22 and CCL20, which act on CCR4^+^ and CCR6^+^ T_reg_, respectively, to induce T_reg_ migration to the TME. In these cases, the accumulation of T_reg_ is associated with reduced patient survival [79,80]. In addition to recruitment of natural T_reg_ to the tumor stroma, TAMs are also involved in the induction of T_reg_ cells in the TME through regulation of Foxp3 via IL-10 and TGF-β signaling [81,82]. However, circulating levels of T_reg_ and TGF-β have been found to be associated with clinical benefit from immune checkpoint inhibitors, pointing to the importance in discerning blood findings from those within the TME [83]. Lastly, TAMs found in human renal cell carcinoma tumor can induce CTLA-4 and Foxp3 expression in T lymphocytes in vivo, further emphasizing the critical role of TAMs in cancer-related inflammation, immunosuppression, and malignant progression [84]. 

Combined with secretion of inhibitory cytokines, chemokines and depleting essential nutrient required for CD8 T cell activation, TAMs achieve subversion of host immune response via interference of CD8 T cell trafficking to the tumor site, and activation of T cell checkpoint blockade by upregulating inhibitory ligands such as PD-L1. Targeting TAMs specifically in the tumor microenvironment to lift the suppressive forces on immune cells particularly CD8^+^ T cells and reinvigorate effector T cells may be an effective way to treat cancer.

## 4. TAM-Targeting Immunotherapies

**Figure 2:**
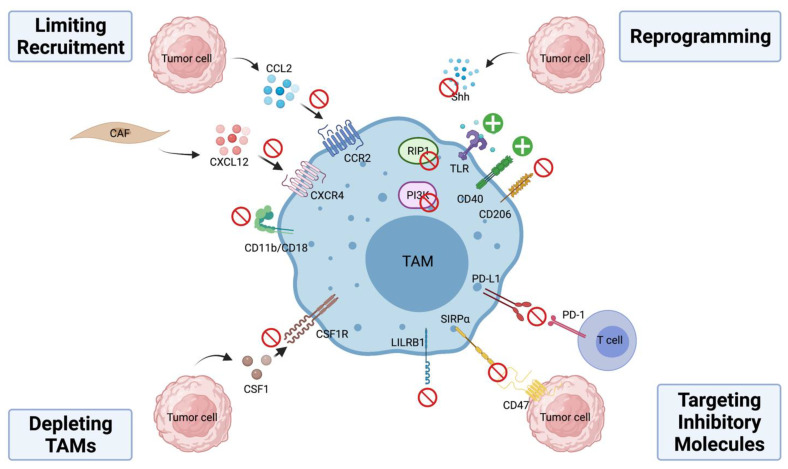
TAM-targeting immunotherapies. Current therapeutic strategies have focused on four major aspects—(1) limiting monocyte recruitment to the tumor site; (2) depleting TAMs; (3) reprogramming TAMs; and (4) targeting inhibitory molecules on TAMs. Inhibition is indicated by red circles while activation is marked by green plus signs. Abbreviations: CAF—cancer-associated fibroblast; CCL2—C-C motif chemokine ligand 2; CCR2—C-C motif chemokine receptor 2; CXCL12—C-X-C motif chemokine ligand 12; CXCR4—C-X-C motif chemokine receptor 4; CD11b/CD18—macrophage-1 antigen; CSF1—colony stimulating factor 1; CSF1R—colony stimulating factor 1 receptor; Shh—sonic hedgehog; RIP1—receptor interacting protein 1; PI3K—phosphoinositide 3-kinase; TLR—Toll-like receptor; CD206—mannose receptor; PD-1—programmed cell death protein 1; PD-L1—programmed death ligand 1; CD47—integrin associated protein; SIRPα—signal-regulatory protein alpha; LILRB1—leukocyte immunoglobulin like receptor B1. Created with BioRender.com.

TAM is an attractive target for cancer immunotherapy characterized by its heavy presence in the tumor stroma across a panel of human malignancies and unique ability to modulate a variety of immune cell functions and non-immune processes in cancer. To overcome the immunosuppressive and pro-tumorigenic functions of TAMs, current therapeutic strategies have focused on four major aspects—(1) limiting monocyte recruitment to the tumor site; (2) depleting TAMs; (3) reprogramming TAMs; and (4) targeting inhibitory molecules on TAMs (Figure 2). Several reviews have highlighted pre-clinical studies and clinical trials that target TAMs in cancer [85,86,87,88]. We will focus on some of the recent successes below and summarize agents studied in clinical trials in Table 1.

### 4.1. Limiting Monocyte Recruitment

Trafficking of monocytes from bone marrow to the tumor site requires the CCL2-CCR2 signaling axis [22]. In various human neoplasia, higher levels of CCL2 are correlated with increased occurrence of metastasis and decreased overall survival [107,108]. In mouse models, inactivation of CCL2-CCR2 signaling achieved by gene knockout or inhibition of CCR2 by small molecule inhibitors reduced tumor growth and metastatic spread [109,110,111]. PF-04136309 is an investigational compound that prevents CCR2 activation and downstream signaling by interfering with CCL2-CCR2 interactions. In a Phase-1b clinical trial comparing a standard chemotherapy regimen FOLFIRINOX (oxaliplatin, irinotecan, leucovorin and fluorouracil) plus PF-04136309 to FOLFIRINOX alone in patients with locally advanced pancreatic ductal adenocarcinoma (PDAC), the addition of PF-04136309 resulted in a reduction in the infiltration of TAMs and T_reg_ and an increase in CD4^+^ and CD8^+^ effector cells, which was correlated with improved treatment response in this group [89]. Recently, Linehan et al. reported that CCX872, a CCR2 specific antagonist showed better overall survival and lower peripheral blood monocyte counts when combined with FOLFIRINOX compared to FOLFIRINOX alone in locally advanced/metastatic PDAC patients [90]. In addition to PF-04136309 and CCX872, Carlumab (CNTO88), a human anti-CCL2 IgG1κ monoclonal antibody (mAb) was evaluated in patients with solid tumors and showed some preliminary anti-tumor activity. However, its clinical efficacy is limited by only transient suppression of CCL2 [91,92,93]. 

Another chemokine, C-X-C motif chemokine ligand 12 (CXCL12) has also been shown to promote macrophage infiltration, accumulation, and survival in tumors [112,113]. In a recent study, Li et al. reported that cancer-associated fibroblast-derived CXCL12 attracts M2-polarized monocytes/macrophages and blocking of C-X-C motif chemokine receptor 4 (CXCR4), the receptor of CXCL12 results in significantly reduced infiltration of pro-tumorigenic TAMs [114]. Mota et al. also observed increased TAM accumulation and tumor progression in a post-sepsis state associated with increased CXCL12/CXCR4 signaling in mice [115]. In a preclinical study using an ovarian cancer model, dual blockade of the CXCL12/CXCR4 and PD-1-PD-L1 signaling cascades effectively reduces tumor burden and prolongs survival in tumor-bearing mice [116]. Multiple clinical studies are currently investigating the effects of CXCR4 blockade in solid tumors with early results [94,95]. Lastly, integrin αMβ2 (CD11b/CD18) is an integrin expressed on myeloid cells that plays a critical role in leukocyte migration and tissue recruitment under inflammatory conditions [117]. Neutralizing monoclonal antibodies against CD11b were shown to reduce recruitment of myeloid cells into squamous cell carcinoma and attenuate tumor growth especially in combination with radiation [118]. Panni et al. utilized a novel strategy by using a partial activator of CD11b, ADH-503 that increases CD11-dependent cell adhesions on the endothelium to prevent subsequent tissue extravasation and found that ADH-503 effectively reduces the number of tumor-infiltrating myeloid cells in murine pancreatic cancer [119]. Collectively, these preclinical and clinical data suggest limiting TAM infiltration is an effective way to curb the immunosuppressive presence of TAMs in various solid tumors. 

### 4.2. Depleting TAMs

CSF-1 is a critical regulator for the differentiation and survival of TAMs; inhibition of CSF1-CSF1R signaling can effectively reduce viability of TAMs [2]. In several preclinical studies, researchers have demonstrated CSF1-CSF1R signaling blockade slowed primary tumor growth, reduced metastatic potential, and improved long-term survival of tumor-bearing mice [120,121,122]. BLZ945 is a potent and selective CSF-1R inhibitor which was shown to attenuate tumor growth by depleting TAMs and enriching CD8^+^ T cells in a murine K14-HPV-16 cervical cancer mouse model [122]. Another agent, PLX3397 selectively inhibits CSF-1R and two other tyrosine kinase receptors KIT and FLT3 [123]. Blockage of CSF-1R with PLX3397 not only improved the efficacy of adoptive cell therapy through inhibition of immunosuppressive macrophage recruitment and activation in murine melanoma but also potentiated the response of xenograft glioblastoma to ionizing radiation (IR) by preventing differentiation and pro-tumoral activation of IR-recruited monocytes in mice [124,125]. In a human phase III study evaluating efficacy of PLX3397 vs. placebo in tenosynovial giant-cell tumor, treatment with PLX3397 showed robust tumor response with improved patient symptoms and functional outcomes [96]. Furthermore, Wesolowski et al. reported that the combination of PLX3397 with paclitaxel showed promising results in advanced solid tumors [97]. Additionally, a neutralizing antibody Emactuzumab (RG7155) against CSF1R reduced macrophage infiltration in mouse models and demonstrated similar therapeutics effects against diffuse-type giant cell tumor in patients [98]. Other neutralizing antibodies against CSF1R—AMG 820 (NCT01444404), IMC-CS4 (NCT01346358), and MCS110 (NCT02807844) are also currently under Phase I/II clinical trials for their efficacy in treatment of advanced solid tumors as a monotherapy [99,100,101]. Many ongoing trials are also investigating the efficacy of CSF1/CSF1R inhibition in combination with traditional chemotherapies or novel immunotherapies in solid tumors as well as hematologic malignancies [126]. 

### 4.3. Reprogramming TAMs

Activation of Toll-like receptors (TLRs) expressed on macrophages leads to M1 polarization through NF-*κ*B signaling. Due to this, TLR agonists have been evaluated in various mouse models for their ability to reprogram TAM functions to destroy tumor cells [127]. Local delivery of a TLR7/8 agonist 3M-052 boosted systemic antitumor immunity by repolarizing TAMs to a M1 NO-producing phenotype and resulted in eradication of murine metastatic melanoma. In addition, combining 3M-052 with antibodies against CTLA-4 and PD-L1 is synergistic in targeting established murine B16.F10 melanoma by rescuing TAM and cytotoxic CD8^+^ T cell tumoricidal functions [128]. Using topical TLR7 agonist imiquimod in patients with skin metastases from breast cancer showed 20% partial response with histologic tumor regression with changes in tumor lymphocytic infiltrate and local cytokines profile [102]. Motolimod, a TLR8 agonist in addition to standard combination chemotherapy and cetuximab was evaluated in a randomized multicenter clinical trial in patients with squamous cell carcinoma of the head and neck. Though the addition did not improve progression-free survival or overall survival, significant benefit was observed in HPV-positive patients, suggesting TLR8 therapy may benefit a subset of patients [103].

Aside from TLR activation, another strategy to activate pro-inflammatory TAMs includes the use of CD40 agonists. Upon activation, CD40, a member of the TNF receptor family that is highly expressed on antigen-presenting cells such as macrophages, drives pro-inflammatory cytokine release and expression of CD80/CD86 to support T cell functions [129,130,131]. Stromnes and colleagues also provided direct evidence that induction of CD40 on TAMs significantly increased the number of proliferating and GramzymeB+ T cells and decreased tumor cell survival in mice [132]. Using a PDAC mouse model, Beatty et al. showed that CD40-activated macrophages rapidly infiltrated tumors and are tumoricidal [133]. Additionally, combining anti-CD40 mAb with CSF-1R inhibitors potently suppressed tumor growth in an autochthonous poorly immunogenic mouse melanoma model. This was correlated with decreased production of MMP9 and CCL17/22 and increased expression of TNF-α, IL-6 and IL-12 in TAMs [134]. Lastly, Diggs et al. found that CD40 agonism sensitizes previously resistant murine intrahepatic cholangiocarcinoma to PD-1 therapy and such effects are mediated through intratumoral macrophages and dendritic cells [135]. The clinical applicability of CD40 agonism was shown in human metastatic pancreatic adenocarcinoma where O’Hara et al. found that APX005M, a CD40 agonistic mAb combined with chemotherapy, with or without nivolumab shows potentially beneficial clinical activity [104]. Several other CD40 agonists—RO7009789 (NCT02665416), SEA-CD40 (NCT02376699), and CP-870893 (NCT01103635) are currently undergoing phase I clinical trials. 

Other exciting advances in efforts to reprogram TAMs in the recent years aim at shifting M2-polarized TAMs into a more phagocytic and pro-inflammatory M1 phenotype. Selective repolarization of TAMs is a promising immunotherapeutic strategy as it would avoid serious side effects such as systemic inflammation, which is harmful to patients, and also disrupt subsequent TAM-derived metabolites that work to foster tumor growth. Wang and colleagues found that using a twin-like core–shell nanoparticles to deliver a TAM repolarization agent IMD-0354 has synergistic antitumor efficacy when combined with traditional sorafenib in mouse models [136]. RP-182, a synthetic molecule that can selectively induce conformational change of the mannose receptor CD206 expressed on TAMs, programs TAMs to an antitumor M1-like phenotype. This results in suppressed tumor growth and extended survival in murine cancer models [137]. Wang et al. reported that inhibition of receptor-interacting serine/threonine protein kinase 1 (RIP1) in TAMs shifted TAMs toward an MHCII^hi^TNFα^+^IFNγ^+^ immunogenic phenotype in a Stat1-dependent mechanism [138]. Additionally, several animal models including PDAC have shown that suppression of PI3Kγ signaling reprograms TAMs to reinstate immune surveillance and hamper tumor progression [139,140]. We also recently demonstrated that inhibition of hedgehog signaling in TAMs increased M1-associated cytokine and chemokine productions while suppressing M2 polarization in mouse hepatoma and Lewis lung carcinoma [24]. This suggests that Shh, which is commonly upregulated in various human cancers, can be an attractive target in cancer immunotherapy for its effects on TAMs in addition to cancer cells. Lastly, others have investigated the possibility of using reprogrammed TAMs as “Trojan Horses” to deliver drugs directly to the tumor site, which aims to promote immune-mediated destruction by inducing tumor microenvironmental changes within the tumor while avoiding the systemic inflammation and toxicity often associated with large scale, indiscriminate administration of treatments [141,142]. This raises exciting possibilities for future novel TAM-targeted therapeutic development. Overall, these data demonstrate reprogramming/repolarizing TAMs is a promising strategy in promoting the tumoricidal effects while minimizing their immunosuppressive functions.

### 4.4. Targeting Inhibitory Molecules on TAMs

Like the role of immune checkpoint receptors PD-1 and CTLA-4 play in suppressing effector T cell functions, the CD47-SIRPα signaling axis inhibits the antibody-dependent phagocytosis of tumor cells by macrophages. CD47, which is highly expressed on the cancer cell surface, engages SIRPα on TAMs to transmit the “no kill” signal. Several CD47-SIRPα antagonists are currently active in clinical trials, including Hu5F9-G4, CC90002, TTI-621 and ALX-148. Hu5F9-G4 and CC90002 are humanized monoclonal antibodies against CD47 while TTI-621 and ALX-148 are SIRPα-IgGFc fusion proteins that can act as decoy receptors [143]. In small cell lung cancer (SCLC), Weiskopf et al. showed that Hu5F9-G2 induced macrophage-mediated phagocytosis of human SCLC cells in vitro. Additionally, in vivo administration of Hu5F9-G4 resulted in suppressed SCLC tumor growth in mice [144]. Another group of researchers demonstrated that Hu5F9-G4 has therapeutic efficacy in vitro and in vivo in patient-derived orthotopic xenograft models of five aggressive pediatric brain tumors [145]. Recently, Upton et al. showed that Hu5F9-G4 combined with trastuzumab can overcome trastuzumab tolerance in HER2+ breast cancer by enhancing antibody-dependent cellular phagocytosis [146]. Petrova et al. also showed that TTI-621 promoted macrophage-mediated tumor killing in a wide array of solid and hematologic malignancies [147]. In clinical trials, Sikic et al. showed that Hu5F9-G4 is well tolerated in patients with advanced solid tumors and Advani et al. demonstrated Hu5F9-G4’s promising antitumor activity in non-Hodgkin’s lymphoma [105,106]. Other clinical trials are currently underway to investigate other antagonists of the CD47-SIRPα pathway.

Another inhibitory molecule expressed by macrophage is leukocyte immunoglobulin-like receptor subfamily B (LILRB). It was found that LILRB1 is upregulated on the surface of TAMs, and its ligand β2-microglobulin expressed by tumor cells can protect LILRB1 from being engulfed. Disruption of this interaction enhances phagocytosis of tumor cells both in vitro and in vivo, suggesting this signal axis is an important regulator of effector functions of TAMs and a potential therapeutic target [148].

It has been reported that many types of human cancers have upregulated PD-L1 expression. In addition, PD-L1 is also commonly expressed on TAMs [149,150,151]. However, the source for PD-L1 in the tumor microenvironment is likely tumor-type specific and varies with different stages of tumor development. Using single cell RNA sequencing data, our laboratory recently showed that PD-L1 expression is mainly found in the myeloid population rather than tumor cells in human hepatocellular carcinoma and intraductal cholangiocarcinoma samples and that the expression of PD-L1 on TAMs is highly dependent on intact hedgehog signaling cascade in these macrophages [27]. Therefore, targeting TAMs and its interactions with cytotoxic T cells by combining hedgehog pathway inhibitor such as vismodegib and PD-1 antibody showed highly synergistic anti-tumor effects in mouse models of hepatoma and Lewis lung carcinoma [24]. This demonstrates the importance of targeting immunoinhibitory molecules on TAMs and the need to explore therapeutic approaches that combine multiple TAM-targeting strategies.

## 5. Conclusions and Future Directions

TAMs are key players in cancer progression via promotion of tumor proliferation, angiogenesis, metastasis, and immune evasion. Disrupting the mutually beneficial, positive feedback loop interactions between TAMs and cancer cells has significant implications in breaking the vicious cycle of progressive immunosuppression in the tumor microenvironment. In this review, we highlighted several mechanisms to target the pro-tumorigenic and immunosuppressive functions of TAMs, including reducing TAM recruitment, depleting or reprogramming TAMs and targeting immunomodulatory molecules on TAMs.

Though promising, the underlying mechanisms of many TAM-targeting therapeutics remain elusive. There is a need for more comprehensive understanding of the induction and functions of TAMs within the tumor microenvironment. Specifically, more investigation is needed for the mechanisms that attract TAMs into the tumor microenvironment and molecules that promote their immunosuppressive phenotype. Current therapies are reliant on targeting several key cascades, such as the CCL2-CCR2, CXCL12-CXCR4, and the CSF1-CSF1R pathways. However, emerging evidence has suggested that the functions of these pathways can be highly context- and tumor subtype-dependent, which may explain the highly variable anti-tumoral effects of these monotherapies. Additionally, as the tumor stroma is a remarkably complex and dynamic environment, further elucidation of novel pathways that may contribute to TAM polarization and immunosuppression is greatly needed. Lastly, our previous reports have highlighted the intimate interactions between TAMs and cytotoxic T cells—not only can TAMs interfere with CD8^+^ T cell trafficking, but they can also directly interact with immune checkpoint inhibitors such as via the PD-L1-PD-1 pathway resulting in T cell exhaustion. Therefore, delineating the mechanisms underlying the upregulation of PD-L1 by TAMs, and defining the role of TAM-derived PD-L1 in CD8 T cell dysfunction within the tumor microenvironment will offer important insights into tumor-TAM-anti-tumor T cell interactions within the tumor microenvironment, and help guide strategies to modulate TAM functions from immunosuppressive to immunostimulating, and reinvigorate cytotoxic T cells and other effector cells. As many therapies that target TAMs are currently being investigated as monotherapies or in addition to traditional chemotherapies, there is a great need to study the potential synergistic effects of TAM-targeting therapies with other effector cells-enhancing strategies to finally overcome the overwhelmingly immunosuppressive tumor microenvironment.

## Figures and Tables

**Table 1:** Summary of TAM-targeting Therapies in Clinical Trials.

Treatment Strategy	Agent Name	Mechanism	Phase	Clinical Trial Number/Reference *
Limiting monocyte recruitment	PF-04136309	CCR2 antagonist	1b	NCT01413022 [89]
	CCX872	CCR2 antagonist	1b	NCT02345408 [90]
	Carlumab	CCL2 antibody	Ib	NCT01204996 [91]
			I	NCT00537368 [92]
			II	NCT00992186 [93]
	LY2510924	CXCR4 antibody	I	NCT02737072 [94]
	Motixafortide	CXCR4 antagonist	IIb	NCT02907099 [95]
Depleting TAMs	PLX3397	CSF-1R antibody	III	NCT02371369 [96]
			Ib	NCT01525602 [97]
	RG7155	CSF-1R antibody	I	NCT01494688 [98]
	AMG 820	CSF-1R antibody	I	NCT01444404 [99]
	IMC-CS4	CSF-1R antibody	I	NCT01346358 [100]
	MCS110	CSF-1 antibody	Ib/II	NCT02807844 [101]
Reprogramming TAMs	Imiquimod	TLR7 agonist	II	NCT00899574 [102]
	Motolimod	TLR8 agonist	II	NCT01836029 [103]
	APX005M	CD40 agonist	I/II	NCT03214250 [104]
	RO7009789	CD40 agonist	I	NCT02665416
	SEA-CD40	CD40 agonist	I	NCT02376699
	CP-870893	CD40 agonist	I	NCT01103635
	IPI-549	PI3Kγ inhibitor	Ib	NCT02637531
			II	NCT03961698
Targeting inhibitory molecules on TAMs	Hu5F9-G4	CD47 antibody	I	NCT02216409 [105]
			I/II	NCT02953509 [106]
			I	NCT03558139
			I/II	NCT02953782
	CC90002	CD47 antibody	I	NCT02367196
	TTI-621	SIRP antibody	I	NCT02663518
			I/II	NCT04996004
	CC-95251	SIRP antibody	I	NCT03783403

* Reference is included when trial has published results.
